# Histone deacetylases inhibitor and RAD51 recombinase increase transcription activator-like effector nucleases-mediated homologous recombination on the bovine β-casein gene locus

**DOI:** 10.5713/ajas.19.0654

**Published:** 2019-10-22

**Authors:** Da Som Park, Se Eun Kim, Deog-Bon Koo, Man-Jong Kang

**Affiliations:** 1 Department of Animal Science, Chonnam National University, Gwangju 61186, Korea; 2 Department of Biotechnology, College of Engineering, Daegu University, Gyeongsan 38453, Korea

**Keywords:** Knock-in, Histone Deacetylases (HDAC) Inhibitor, Homologous Recombination (HR), RAD51 recombinase (RAD51), Transcription Activator-like Effector Nucleases (TALEN)

## Abstract

**Objective:**

The efficiency of the knock-in process is very important to successful gene editing in domestic animals. Recently, it was reported that transient loosening of the nucleosomal folding of transcriptionally inactive chromatin might have the potential to enhance homologous recombination efficiency. The objective of this study was to determine whether histone deacetylases (HDAC) inhibitor and RAD51 recombinase (RAD51) expression were associated with increased knock-in efficiency on the β-casein (*bCSN2*) gene locus in mammary alveolar-large T antigen (MAC-T) cells using the transcription activator-like effector nucleases (TALEN) system.

**Methods:**

MAC-T cells were treated with HDAC inhibitors, valproic acid, trichostatin A, or sodium butyrate for 24 h, then transfected with a knock-in vector, RAD51 expression vector and TALEN to target the *bCSN2* gene. After 3 days of transfection, the knock-in efficiency was confirmed by polymerase chain reaction and DNA sequencing of the target site.

**Results:**

The level of HDAC 2 protein in MAC-T cells was decreased by treatment with HDAC inhibitors. The knock-in efficiency in MAC-T cells treated with HDAC inhibitors was higher than in cells not treated with inhibitors. However, the length of the homologous arm of the knock-in vector made no difference in the knock-in efficiency. Furthermore, DNA sequencing confirmed that the precision of the knock-in was more efficient in MAC-T cells treated with sodium butyrate.

**Conclusion:**

These results indicate that chromatin modification by HDAC inhibition and RAD51 expression enhanced the homologous recombination efficiency on the *bCSN2* gene locus in MAC-T cells.

## INTRODUCTION

The knock-in of large DNA fragments has been restricted by technical difficulties attributed in part to the low efficiency of homologous recombination (HR) in cells. Recently, it was demonstrated that use of zinc finger nucleases (ZFN), transcription activator-like effector nucleases (TALEN), and the clustered regularly interspaced short palindromic repeats/CRISPR-associated 9 (CRISPR/Cas9) system could enhance the knock-out or knock-in efficiency in mammalian cells compared to traditional methods [1–4].

If double-strand breaks (DSBs) occur at specific loci cut by gene scissors, such as ZFN, TALEN, and CRISPR/Cas9, DSBs are repaired in the cells by HR or non-homologous end joining (NHEJ) [1,3,5,6]. While NHEJ gives rise to deletions and insertions (indels) at the target site, HR utilizes a donor DNA template (knock-in vector) for precise repair at the target site [1,3,5,6].

Although knock-in efficiency in the cells is largely-dependent on a variety of factors, such as size of the HR arm, transfection methods, marker selection, and target gene locus, the β-casein locus of somatic cells has been targeted using ZFN, TALEN, and CRISPR/Cas9 with an efficiency of 4.5% to 80% using transfection and selection strategies [7–10]. Previously, it has been suggested that knock-in efficiency was increased by regulation of cell cycle [11] or the NHEJ and HR pathways [12,13]. Treatment of cells with SCR7 pyrazine (an inhibitor of NHEJ) decreased NHEJ and enhanced HR [12]. And RAD51 recombinase (RAD51) overexpression induced knock-in efficiency in the cells [13].

However, the state of the eukaryotic chromatin has also been shown to influence the knock-in efficiency by regulation of CRISPR/Cas9 binding at the specific gene locus [14]. Recent studies have indicated that an open chromatin state with an active transcription site was stimulated more by CRISPR/Cas9 than heterochromatin [15]. Takayama et al [16] reported that human embryonic stem cells/Induced pluripotent stem cells (ES/iPS cells) were bi-allelically targeted by TALEN and an histone deacetylases (HDAC) inhibitor and/or RAD51 expression with an efficiency of 4% to 38% at various gene loci.

Enhancement of the knock-in efficiency of large fragments is required to produce recombinant proteins through regulation of the endogenous gene regulatory DNA sequence containing the enhancer and the promoter in the mammal gland system of the cell or animal. In this study, our aim was to develop a method with high knock-in efficiency, regardless of the chromatin state of the target site. In order to enhance knock-in efficiency, we investigated whether HDAC inhibition and/or expression of RAD51 increased knock-in efficiency at the β-casein locus in mammary alveolar-large T antigen (MAC-T) cells.

## MATERIALS AND METHODS

### Construction of knock-in vector

**Figure 1:**
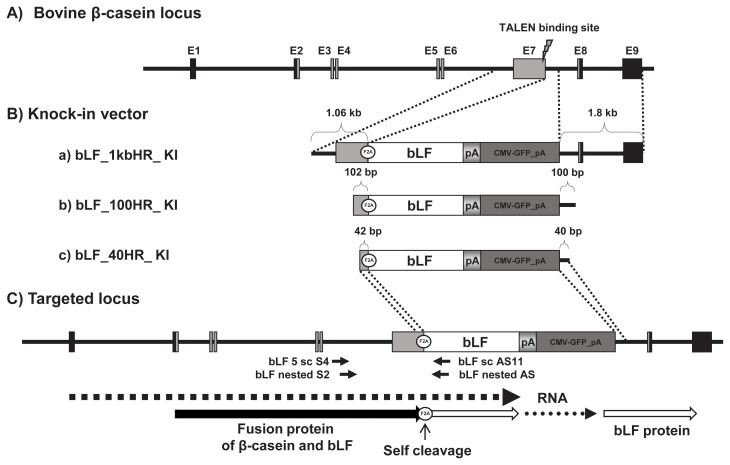
The knock-in strategies of knock-in vectors on bovine β-casein exon 7 locus for expression of bovine lactoferrin. A) Genomic structure of the bovine β-casein gene locus. B) Three knock-in vectors: a) bLF_1kbHR, b) bLF_100HR, and c) bLF_40HR. These targeting vectors used cytomegalovirus-green fluorescent protein (*CMV-GFP*) gene as a positive selection marker. C) Targeted locus of the knock-in vector by homologous recombination. The polymerase chain reaction primer pairs used for detecting homologous recombination events are shown by arrows and names.

In order to construct a bovine lactoferrin (*bLF*) 1kbHR knock-in vector containing 1 kb 5′ and 1.8 kb 3′ homologous arm, we modified the pBSK(-)m_tEndo_enhanced green fluorescent protein (EGFP) knock-in vector from Kim et al [17]. First, the *bLF* cDNA was amplified from a mammary gland by reverse transcription polymerase chain reaction (RT-PCR) using the *bLF* KpnI S primer (GGTACCATGAAGCTCTT CCTCCCCGCCCTGCTGT) and the *bLF* XbaI AS primer (TCTAGATTACCTCGTCAGGAAGGCGCAGGCTTC). Then, the KpnI site (GGTACC) in *bLF* cDNA was replaced with GGTAGC by PCR mutagenesis using the KpnI restriction enzyme for DNA sub-cloning. This *bLF* cDNA (KpnI-XbaI fragment) was sub-cloned into the KpnI-XbaI sites of the pBSK(-)m_tEndo_EGFP knock-in vector to produce the *bLF*_1kbHR knock-in vector. To construct the *bLF*_100HR and *bLF*_40HR knock-in vector, we amplified each knock-in vector by PCR using a 100HR primer (sense, GCGCGGCC GCAAAGCAGTGCCCTATCC; anti-sense, GCGTCGACA GCTATGCTTATTTTGGAAC) and a 40HR primer (sense, GCGCGGCCGCCCTGTACTAGGTCCTGTCC; anti-sense, GCGTCGACGAATATCATACAAACATCAG) and the *bLF* _1kbHR knock-in vector as a template (Figure 1B). These knock-in vectors were digested with the NotI restriction enzyme to linearize them prior to transfection.

### Cell culture and electroporation

Cell culture and transfection of the knock-in vector were conducted according to previously reported methods [17]. The MAC-T cells were cultured in growth medium: Dulbecco’s modified Eagle’s medium (DMEM, HyClone Co., Logan, UT, USA) containing 5% fetal bovine serum (Atlas, Ft. Collins, CO, USA) and 1% penicillin/streptomycin (HyClone, USA). MAC-T cells at 70% confluency were treated with an HDAC inhibitor, valproic acid (VPA, 10 μM), trichostatin A (TCA, 100 nM), or sodium butyrate (SB, 10 μM), for 24 hours before electroporation. For electroporation, the MAC-T cells treated with HDAC inhibitor were harvested by treatment with 0.25% trypsin-ethylenediaminetetraacetic acid and resuspended in Ham’s F10 medium (HyClone Co., USA) at a density of 1.25×10^7^ cells per mL of medium. The cell suspension (400 μL) was electroporated with 1.5 μg of the linearized knock-out vector and 1.5 μg each of TALEN expression plasmid [16] with or without 1 μg RAD51 expression vector in a 4-mm cuvette using four 1-ms pulses and 250-V capacitive discharges from a BTX Electro-cell Manipulator (ECM 2001, BTX, Holliston, MA, USA). After electroporation, the cells were cultured for 48 h in a culture dish. Genomic DNA was isolated from the transfected cells using the G-DEX IIc Genomic DNA extraction Kit For Cells and Tissues (iNtRON, Seongnam, Korea) for analysis of knock-in by PCR.

### Western blot analysis of histone deacetylases 2

Protein from the cells was isolated using the PRO-PREP protein extraction kit (iNtRON, Korea) according to the manufacturer’s instructions. For Western blots, 50 μg protein was separated on 10% sodium dodecyl sulfate–polyacrylamide gel electrophoresis gels and transferred to polyvinylidene fluoride membranes (Bio-Rad Co., Hercules, CA, USA). The membranes were blocked with 5% skim milk and 0.05% Tween 20 in Tris-buffered saline (TBS) overnight at 4°C, then blotted with a mouse monoclonal anti-HDAC2 antibody (1:1,000 dilution, sc-9959; Santa Cruz Biotechnology, Dallas, TX, USA) or a mouse anti-β-actin antibody (1:1,000 dilution, sc-47778; Santa Cruz Biotechnology, USA) overnight at 4°C. After three washes with 0.05% Tween 20 in TBS, the membranes were incubated for 2 h with a mouse immunoglobulin G (IgG) kappa binding protein (m-IgGκ BP) conjugated to horseradish peroxidase (1:2,000 dilution, sc-2005; Santa Cruz, USA). The membranes were then washed three times with 0.05% Tween 20 in TBS. The protein bands were detected by incubation with EZ-Western Lumi Pico or Femto kit (DOGEN, Seoul, Korea). The bands were visualized using an infrared imaging system (LI-COR Odyssey Fc Imaging System; LI-COR Biosciences, Lincoln, NE, USA). The Western blots were repeated three times and the band density was calculated using the UN-SCAN-IT gel (Silk Scientific Inc., Orem, UT, USA) program and are presented as bar graphs.

### Expression analysis of bovine β-casein gene by real-time polymerase chain reaction

Total RNA was isolated from MAC-T cells treated with HDAC inhibitors using the RNeasy mini kit (Qiagen, Hilden, Germany). The total RNA was then treated with DNase I, then reverse transcription was performed at 42°C for 90 min using 5 μg of total RNA, random primers, and Superscript II RNase H-Reverse transcriptase (Invitrogen, Carlsbad, CA, USA). Comparative real-time PCR was performed using 20 μL of reaction solution containing 10 μL of TOPreal qPCR 2X PreMIX (Enzynomics, Daejeon, Korea), 40 ng of cDNA and each primers (bCSN2-F[10 pmole]; AGCCATGAAGGTCCTCA TCC, bCSN2-R[20 pmole]; CTCACTGCTTGAAAGGC TTT). Each reaction was conducted in triplicate and run in a Stratagene Mx3000P Real-Time PCR system (Agilent Technologies, Inc., Santa Clara, CA, USA). The thermal cycling conditions were as follows; 40 cycles of denaturation at 95°C for 30 s, annealing at 55°C for 10 min, and extension at 72°C for 1 min. Next, the mRNA levels were corrected using the transcription level of the mouse riposomal protein lateral stalk subunit P0 gene as an internal standard.

### Analysis of knock-in efficiency by polymerase chain reaction

Genomic DNA was isolated from cells using the G-DEX IIc Genomic DNA Extraction Kit For Cells and Tissues (iNtRON, Korea) and was used as the PCR template to identify the knock-in. First PCR was conducted, then nested PCR was performed to confirm the knock-in efficiency in the cells. The first PCR was performed in a 25-μL reaction mixture containing 50 ng genomic DNA, 0.1 M each of *bLF 5sc S4* sense (GCCTGCTTGCCGAATATCATGGTGGAAAAT) for regions outside the 5′ arm, and *bLF sc AS11* antisense (GGGGAGAGGAAGGGAGAAGCTTAATAGTGG) primers, and TAKARA EX Taq (Takara Co., Tokyo, Japan). Amplification was performed by 20 cycles of denaturation at 98°C for 10 s, annealing at 60°C for 30 s, and extension at 72°C for 1.5 min. Nested PCR was conducted using 0.05 μL of the first PCR products as a template with *bLF nested S2* (GAGAGAAGTGAGGTACAGGACAATTGAG), *bLF nested AS* antisense (ATGGTACACCATCGAACGTTTTT CCTCG) primers and TAKARA EX Taq (Takara Co., Japan) as follows: denaturation at 98°C for 10 s, annealing at 60°C for 30 s, and extension at 72°C for 1.5 min (25 cycles from denaturation to extension), and final extension at 72°C for 5 min. The PCR products were confirmed by electrophoresis on a 0.8% agarose gel. In order to identify the precision of the knock-in, the PCR products were ligated into a pGEM-T Easy Vector (Promega, Madison, WI, USA) to facilitate the DNA sequencing process. The DNA sequences were analyzed by the SolGent Co. and DNA analysis was performed with GENETYX software version 4.0.

### Statistical analysis

All the results were expressed as mean±standard error of mean. Statisxal differences were evaluated using GraphOad PRISM ver. 5 software (GraphPad software, La Jolla, CA, USA). mRNA expression was compared using one-way analysis of variance, followed by Dunnett’s multiple comparison test. The “t test” was used to examine between-group differences. A level of p<0.05 was considered statistically significant.

## RESULTS

### Knock-in vector and expression model of *bLF* protein in the bovine β-casein gene locus using the F2A self-processing peptide

The diagram of the knock-in vector for expression of the bovine β-casein fused lactoferrin gene in the mammary gland is shown in Figure 1. The knock-in vector consisted of the 5′-homologous arm, the furin-2A (F2A) sequence, *bLF* cDNA, bovine growth hormone poly A signal, the cytomegalovirus-green fluorescent protein (*CMV-GFP*) gene, and the 3′-homologous arm. *CMV-GFP* was used as a positive selection marker to visualize expression of the knock-in vector in the cells. In this study, the F2A-fused *bLF* cDNA was introduced into the exon 7 locus of the bovine β-casein (*bCSN2*) gene. To determine knock-in efficiency based on the length of the homologous arm in the knock-in vector, we constructed three different knock-in vectors (Figure 1B). The *bLF* mRNA in the knock-in vectors can be expressed together with the β-casein sequence from exon 1 and 7 owing to their linkage through the F2A sequence. If the knock-in vector underwent homologous recombination in the bovine *bCSN2* gene locus, then *bLF* is expressed under control of the bovine β-casein promoter and all gene-regulatory sequences, including an enhancer. This fusion protein is probably cleaved between the β-casein and F2A self-cleavage sites. Moreover, the F2A peptide is believed to disrupt translation and impair normal peptide bond formation between the proline and glycine of F2A, without affecting translation of the bLF protein. According to this model, *bLF* mRNA is probably translated with the bovine β-casein sequence and the F2A peptide. After that, the bLF protein can be cleaved to remove the bovine β-casein sequence and F2A peptide from the fusion protein (Figure 1C).

### HDAC2 protein and β-casein mRNA expression in MAC-T cells treated with HDAC inhibitors

**Figure 2:**
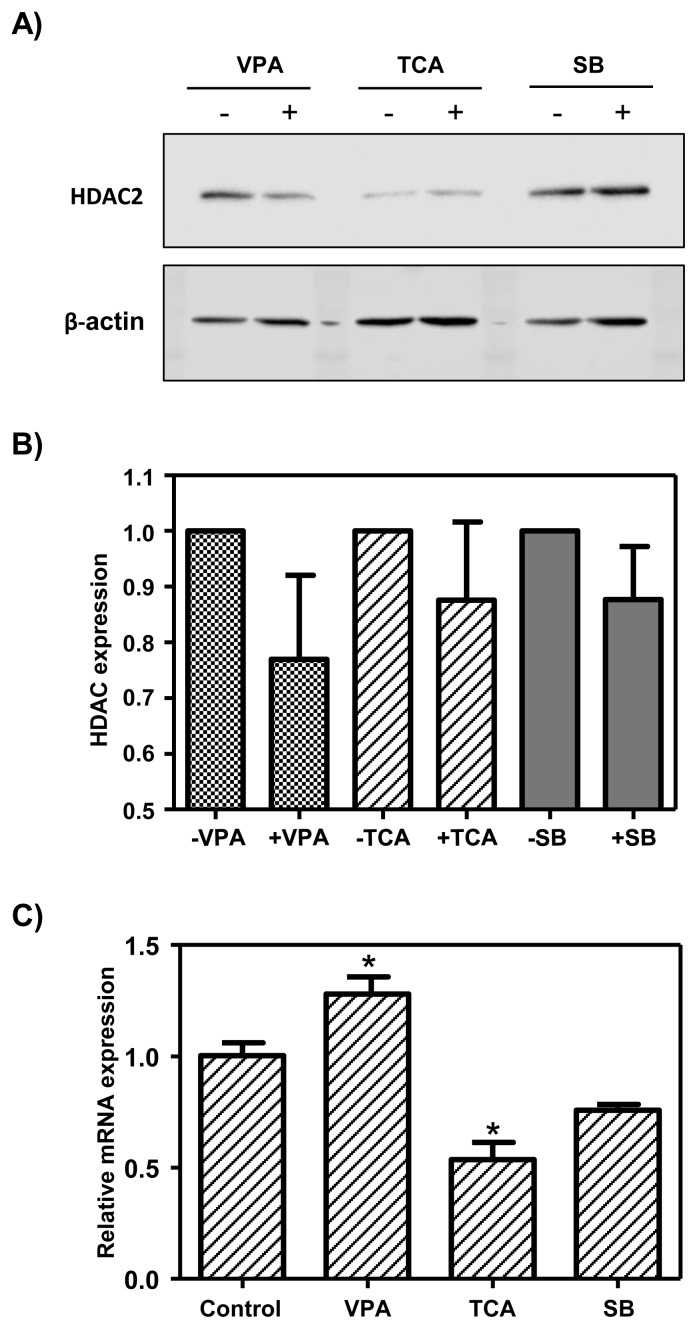
Analysis of gene expression of HDAC inhibitor-treated MAC-T cells. A) Western blot analysis of HDAC2 protein expression in HDAC inhibitor-treated MAC-T cells. B) The graph represents the densitometric quantification of protein bands. Protein bands were normalized by calculating the HDAC2/β-actin ratio. HDAC, histone deacetylases; MAC-T, mammary alveolar-large T antigen; VPA, valproic acid; TCA, trichostatin A; SB, sodium butyrate. Values are expressed as the mean±standard error of triplicate tests. The “paired t- test” was used to examine between treated and not treated HDAC inhibitor, respectively. C) The mRNA expression level of bCSN2 in HDAC inhibitor-treated MAC-T cells by real-time polymerase chain reaction. Values are expressed as the mean±standard error of triplicate tests. * p<0.05 vs control by analysis of variance and Dunnett’s multiple comparison test. Control: MAC-T cell not treated HDAC inhibitor.

To determine whether HDAC inhibitors, such as VPA, TCA, and SB, decreased HDAC2 protein in the MAC-T cells, we conducted Western blot analysis. The results showed that expression of the HDAC2 protein was reduced about 25% by VPA treatment and was decreased about 15% by TCA treatment. HDAC2 protein expression was not decreased by SB treatment (Figure 2A, B). However, the expression of HDAC2 protein was slightly different in the valproic and TCA treatment groups, but there was no statistical difference. HDAC inhibitors are known to induce gene expression by altering the chromatin structure. Therefore, the mRNA expression of the *bCSN2* gene in MAC-T cells treated with HDAC inhibitors was confirmed by real-time PCR. The expression bCSN2 mRNA was significantly higher in MAC-T cells treated with VPA than in the control cells. However, when MAC-T cells were treated with TCA, bCSN2 mRNA expression was decreased than in the control cells. Also, bCSN2 mRNA expression was not different than in the control cells when MAC-T cells were treated with SB (Figure 2C).

### Treatment with HDAC inhibitors and ectopic expression of RAD51 increase TALEN-mediated homologous recombination on the bovine β-casein gene locus

**Figure 3:**
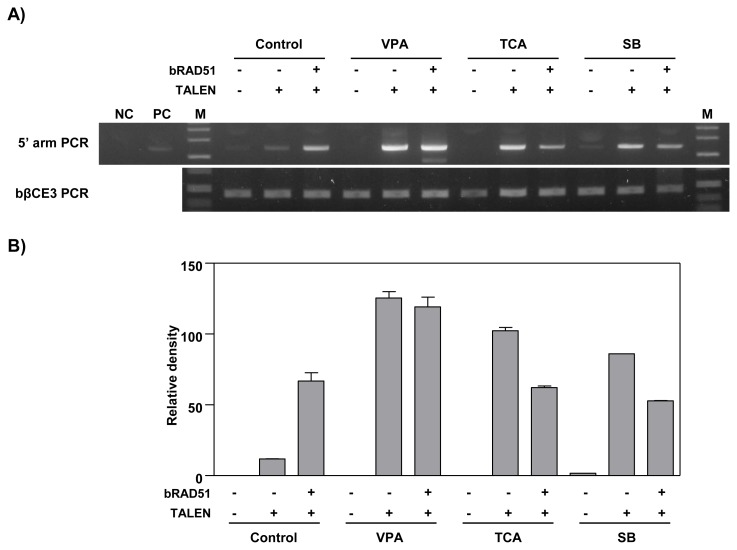
PCR analysis of gene targeting efficiency by treatment with HDAC inhibitors and over-expression of RAD51 in MAC-T cells. A) Agarose gel electrophoresis of knock-in DNA band. MAC-T cells were treated with 10 μM VPA, 100 nM TCA, or 10 μM SB for 24 h and transfected with the bLF_1kbHR knock-in vector with TALEN or RAD51 expression plasmid. After 3 days of transfection, the knock-in efficiency was confirmed by PCR. The top panel shows the 5′ arm PCR. The bottom panel shows the bovine β-casein gene exon 3 internal PCR showing that the same amount of genomic DNA was used. PCR, polymerase chain reaction; HDAC, histone deacetylases; RAD51, RAD51 recombinase; MAC-T, mammary alveolar-large T antigen; VPA, valproic acid; TCA, trichostatin A; SB, sodium butyrate; TALEN, transcription activator-like effector nucleases; NC, negative control (a mixture of bovine genomic DNA and bLF knock-in vector); PC, positive control; M, size marker (1 kb ladder). B) The graph represents the densitometric quantification of DNA bands.

To investigate the effect of HDAC inhibitors and *RAD51* gene expression on the gene targeting efficiency of the *bCSN2* gene locus, a bLF_1kbHR knock-in vector was introduced into MAC-T cells. The knock-in efficiency analyzed by PCR is presented in Figure 3. After the knock-in vector with TALEN alone was transfected into MAC-T cells not treated with an HDAC inhibitor, the knock-in efficiency was confirmed by PCR in all experimental groups, control, VPA, TCA, and SB treated cells. High knock-in efficiency was observed in VPA -treated cells. Also, the knock-in efficiency was higher in cells treated with HDAC inhibitors than in control cells not treated with an HDAC inhibitor. In addition, when the cells were transfected with the knock-in vectors, TALEN and RAD51 expression vector, the knock-in efficiency was increased by VPA. Similar knock-in efficiency was observed in control cells and cells treated with TCA or SB. Overall, the knock-in efficiency was increased in MAC-T cells treated with VPA in the TALEN-mediated gene targeting process. The three HDAC inhibitors increased the knock-in efficiency in MAC-T cells also induced for the ectopic expression of the *RAD51* gene (Figure 3).

**Figure 4:**
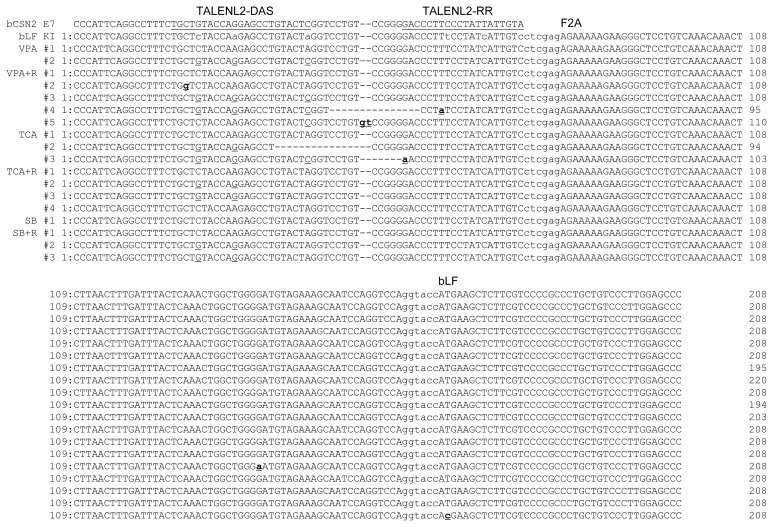
Sequence analysis at the transcription activator-like effector nucleases (TALEN) binding site and the 5′ junction of the genome integrated with the furin-2A (F2A) and bovine lactoferrin (bLF) sequences of the knock-in vector. Underlines indicate TALEN binding site on the genomic sequence. Lower case in the bLF KI indicates the mutated TALEN binding site in the knock-in vector. The underlined single letter shows the recovered nucleotide of the mutated TALEN binding site in the knocked-in vector. Bold underlined lower case indicates a mutation in the knock-in site. F2A indicates the start site of the F2A sequence and bLF indicates the start site of the bLF sequence. ctcgag is the XhoI restriction enzyme site. ggtacc is the BamHI restriction enzyme site.

In order to confirm the knock-in precision, the PCR products were sequenced and the target site and TALEN binding site were analyzed (Figure 4). The point mutation in the left TALEN binding site (TALENL2-DSA) in the knock-in vector was replaced with the original sequence of the target clone (VPA #2 and VPA+R #3). Deletion or insertion of the TALEN cleavage site was confirmed in the VPA+R #4, VPA+R #5, TCA #2, and TCA #3 clones. These clones encoded incorrect proteins confirmed by analysis of the protein-encoding DNA sequences using Genetyx version 4 (data not show). Also, although the point mutation in the left TALEN binding site (TALENL2-DSA) was replaced, precise knock-in was identified in the SB+R #2 and #3 clones. However, a precise knock-in containing the knock-in vector sequence was confirmed in the VAP #1, VPA+R #1, TCA #1, TCA+R #1, SB #1, and SB+R #1 clones. These results indicate that these clones may synthesize the correct proteins of β-casein fused F2A peptide and *bLF*. However, in the TSA + R # 4 clone, G in the F2A nucleotide sequence, which is not a TALEN recognition site, was substituted with A, resulting in the substitution of aspartic acid with asparagine in the protein (data not show). But, in the SB+R # 3 clone, T in the ATG position of the *bLF* gene was replaced with C, indicating that the initiation codon, methionine, was replaced with threonine (data not show). These results indicated that the knock-in efficiency was increased by TALEN, HDAC inhibitors, and RAD51 expression, whereas the point mutation was induced on the target site and knock-in vector.

**Figure 5:**
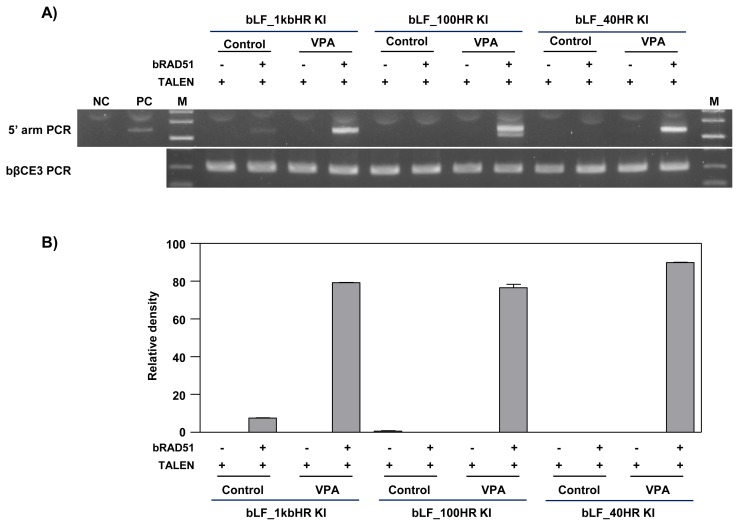
PCR analysis of gene targeting efficiency according to homologous arm length of the knock-in vector in valproic acid-treated MAC-T cells. A) Agarose gel electrophoresis of knock-in DNA band. MAC-T cells were treated with 10 μM valproic acid for 24 h and transfected with bLF_1kbHR, 100HR, or 40HR_knock-in vector and TALEN or RAD51 expression vectors. After 3 days of transfection, the knock-in efficiency was confirmed by PCR. The top panel shows the 5′ arm PCR. The bottom panel shows bovine β-casein gene exon 3 internal PCR showing that the same amount of genomic DNA was used. PCR, polymerase chain reaction; MAC-T, mammary alveolar-large T antigen; TALEN, transcription activator-like effector nucleases; RAD51, RAD51 recombinase; VPA, valproic acid; NC, negative control (a mixture of bovine genomic DNA and bLF knock-in vector); PC, positive control; M, size marker (1kb ladder). B) The graph represents the densitometric quantification of DNA bands.

**Figure 6:**
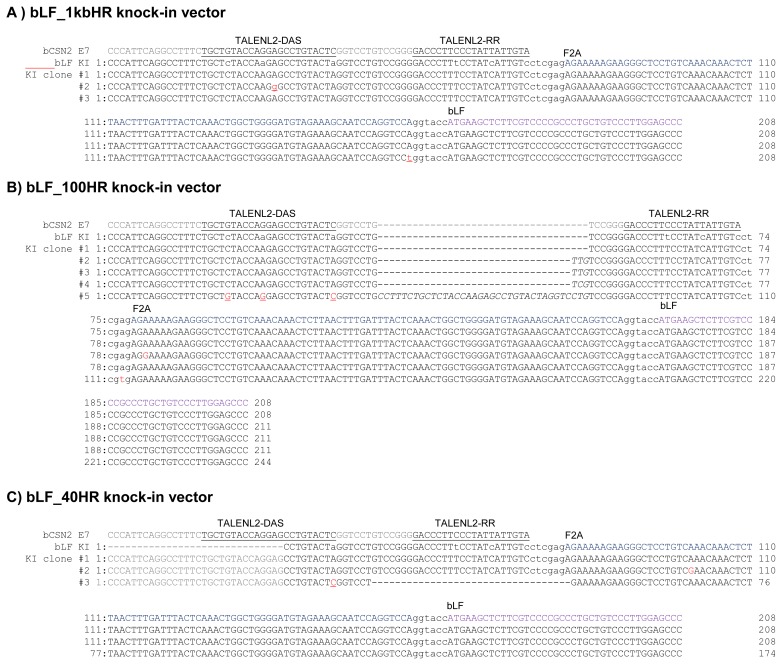
Nucleotide sequence analysis of TALEN target site in knocked-in MAC-T cells transfected with three kinds of vectors harboring different homologous arm lengths A) Clones from cells transfected with the bovine lactoferrin (bLF)_1kbHR knock-in vector. B) Clones from cells transfected with the bLF_100HR knock-in vector. C) Clones from cells transfected with the bLF_40HR knock-in vector. Underlines indicate the TALEN binding site on the genomic sequence. Lower case in the bLF KI indicates the mutated TALEN binding site in the knock-in vector. The underlined single letter shows the recovered nucleotide on the mutated TALEN binding site in the knocked-in vector. Bold underlined lower case indicates mutations in the knock-in site. Upper case italics indicate insertion of nucleotide upon cleavage by TALEN. Furin-2A (F2A) indicates the start site of the F2A sequence and bLF indicates the start site of the bLF sequence. ctcgag is the XhoI restriction enzyme site. ggtacc is the BamHI restriction enzyme site. TALEN, transcription activator-like effector nucleases; MAC-T, mammary alveolar-large T antigen.

To examine the homologous arm length of knock-in vector required for TALEN-mediated knock-in, we tested three knock-in vectors bearing homologous arms from 40 bp, 100 bp, and 1 kb in length in MAC-T cells treated with VPA and induced for *RAD51* gene expression. When compared to the control group not treated valproic aid, knock-in efficiency was elevated in MAC-T cell treated with VPA and co-transfected with TALEN and RAD51, regardless of the homologous arm length of the vector (Figure 5). The precise knock-in was confirmed by sequencing and analysis of the TALEN binding site (Figure 6). In MAC-T cells transfected with the bLF_ 1kbHR_GFP knock-in vector, precise knock-in was identified, although some clones (Clone #2 and 3) showed a point mutation (A→G in clone #2 and A→T in clone #3). However, when the bLF_100HR_GFP knock-in vector was introduced into MAC-T cell, four out of five clones showed insertion of 3 or 36 bp on the TALEN FokI cleavage site. These clones may synthesize incorrect amino acids from the targeted locus. In MAC-T cells transfected with the bLF_40HR_GFP knock-in vector, precise knock-in was identified in two clones (Clone #1 and 2) out of three, and one clone (Clone #3) showed a 34 bp deletion on the TALEN FokI cleavage site. These results indicate that these knock-in methods can increase the knock-in efficiency on the *bCSN2* gene locus but can also increase mutations at the target site of TALEN.

## DISCUSSION

In this study, the knock-in efficiency on the *bCSN2* gene locus was increased by TALEN-mediated homologous recombination in MAC-T cells treated with HDAC inhibitors, which inhibited HDAC, and ectopic expression of the *RAD51* gene, which encodes the recombinase that assists in the repair of DNA DSBs.

These efficient knock-in methods can be exploited to facilitate correction of diseased genes and overexpression of transgenes on specific gene loci. Homologous recombination in cells has been reported to occur most frequently during the S phase and less frequently during the G2/M phase [18]. Various knock-in methods have been developed over the past decade using ZFN, TALEN, and CRISPR/Cas9 [1,2,5,6]. These methods are more efficient than conventional methods, but their knock-in efficiency has been reported to differ depending on the vector structure, target gene locus, and cell type [19–21]. Some researchers are investigating ways to improve knock-in efficiency using cell cycle synchronization techniques [11,22]. Zhang et al [22] showed that the combined use of CCND1 and nocodazole enhanced the double knock-in efficiency to up 30% in iPSCs. Lin et al [11] reported that knock-in methods using cell cycle synchronization increased Cas9 RNP-mediated HDR in HEK293T cells, human primary neonatal fibroblasts, and human embryonic stem cells.

Furthermore, some studies have reported that CRISPR/Cas9- and TALEN-mediated knock-in efficiency could be increased by suppression of the NHEJ pathway in cells and zygotes [12,13]. Song et al [13] reported that treatment with RAD51-stimulatory compound 1, an HDR enhancer, and RAD51 mRNA injection synergistically increased knock-in efficiency in rabbit embryos. The efficiency of gene editing was reported to be influenced by the chromatin state of the target site of ZFN, TALEN, and CRISPR/Cas9 [14]. Moreover, it has been reported that RAD51 overexpression and HDAC inhibition by VPA treatment in human ES/iPS cells increased the bi-allelic knock-in efficiency [16]. Takayama et al [16] reported that when cells were treated with VPA or RAD51 alone, the knock-in efficiency was 2.6 times higher in untreated cells or cells treated with RAD51 alone, and increased about 4 times when the cells were co-treated with VPA and RAD51. In this study, we identified an increased knock-in efficiency on the *bCSN2* gene locus in MAC-T cells treated with VPA, TCA, and SB. Additional *RAD51* gene expression in cells treated with HDAC inhibitors induced more stable knock-in efficiencies. These results indicate that HDAC inhibitor treatment and ectopic expression of *RAD51* gene induced knock-in efficiency in MAC-T cells. In addition to VPA, TCA, and SB also increased knock-in efficiency.

In order to analyze the knock-in efficiency dependent on the homologous arm length in the knock-in vector, we introduced knock-in vectors with different lengths into MAC-T cells overexpressing *RAD51* gene and treated with HDAC inhibitors. No difference in knock-in efficiency with different arm lengths was found. In conventional gene targeting in ES cells, the length of the homologous arm is recommended to be at least 5 to 8 kb, and the gene targeting efficiency was reduced when the length of the homologous arm was less than 1 kb [23]. However, using ZFN, TALEN, and CRISPR/Cas9 successful genomic editing from 100 bp to several kb of homologous regions has been reported [24–27]. It has also been reported that single-strand donor DNA (single strand knock-vector) can result in higher knock-in efficiency than double-strand donor DNA (double strand knock-in vector) [8]. Hisano et al [20] reported that knock-in vectors harboring 10 to 40 bp homologous arms could be introduced into a target locus using CRISPR/Cas9-mediated knock-in in the zebrafish embryo. In addition, when the CRISPR/Cas9 system was used for mouse zygotes, homologous recombination of the knock-in vector occurred at an efficiency of 2% to 12% when a knock-in vector containing a 500 bp homologous arm was used, but homologous recombination did not occur when a knock-in vector containing 60 to 250 bp homologous arms was used [28]. However, Zhang et al [22] reported that the knock-in efficiency increased according to the length of the homologous arms in 293 T cells by the double-cut donor system using CRISPR/Cas9. In contrast, in this study, we have shown that the knock-in efficiency did not differ according to the length of the homologous arm. These results indicate that homologous arm length did not affect the knock-in efficiency of this system using HDAC inhibitors and overexpression of RAD51. In this study, when the precise knock-in site was analyzed by sequencing, some clones had point mutations, small insertions, or deletions in the target site of TALEN. These results are probably due to cut TALEN binding site introduced point mutations on the knock-in vector by TALEN.

In conclusion, treatment with HDAC inhibitors and ectopic expression of RAD51 in MAC-T cells increased knock-in efficiency on the *bCSN2* gene locus in this study. Although the molecular function of co-regulation by HDAC inhibition and RAD51 overexpression in homologous recombination has not been clarified, it has been suggested that HDAC inhibitor and RAD51 enhanced homologous recombination by chromatin modification and recombination enhancement. When VPA was added to the cells, there was no change in the knock-in efficiency associated with the length of the homologous arm. Therefore, future studies are necessary to further clarify the molecular mechanism of the gene targeting by HDAC inhibitors and RAD51. Improvement of the knock-in efficiency on the *bCSN2* gene locus using HDAC inhibitor treatment and overexpression of RDA51 may contribute to the production of recombinant proteins from the knock-in on the *bCSN2* gene.
